# Interprofessional communication in a psychiatric outpatient unit – an ethnographic study

**DOI:** 10.1186/s12912-023-01446-y

**Published:** 2023-08-25

**Authors:** Ingela Rudberg, Annakarin Olsson, Charlotta Thunborg, Martin Salzmann-Erikson

**Affiliations:** 1 Department of Health and Caring Sciences, Faculty of Health and Occupational Studies, University of Gävle, Gävle, SE-801 76 Sweden; 2 Division of Clinical Geriatrics, Department of Neurobiology, Care Sciences and Society, Karolinska Institutet, Stockholm, Sweden; 3 Department of Health and Welfare, Mälardalen University, Västerås, Sweden

**Keywords:** Psychiatric outpatient care, Interprofessional communication, Focused ethnography, Code of conduct

## Abstract

**Background:**

Communication in healthcare has been extensively studied, but most research has focused on miscommunication and the importance of communication for patient safety. Previous research on interprofessional communication has mainly focused on relationships between physicians and nurses in non-psychiatric settings. Since communication is one of the core competencies in psychiatric care, more research on interprofessional communication between other clinicians is needed, and should be explored from a broader perspective. This study aimed to explore and describe interprofessional communication in a psychiatric outpatient unit.

**Method:**

During spring 2022, data consisting of over 100 h of fieldwork were collected from observations, formal semi-structured interviews and informal conversations inspired by the focused ethnography method. Data was collected at an outpatient unit in central Sweden, and various clinicians participated in the study. The data analysis was a back-and-forth process between initial codes and emerging themes, but also cyclical as the data analysis process was ongoing and repeated and took place simultaneously with the data collection.

**Results:**

We found that a workplace’s history, clinicians´ workload, responsibilities and hierarchies influence interprofessional communication. The results showed that the prerequisites for interprofessional communication were created through the unit’s code of conduct, clear and engaging leadership, and trust in the ability of the various clinicians to perform new tasks.

**Conclusion:**

Our results indicate that leadership, an involving working style, and an environment where speaking up is encouraged and valued can foster interprofessional communication and respect for each other´s professional roles is key to achieving this. Interprofessional communication between different clinicians is an important part of psychiatric outpatient work, where efficiency, insufficient staffing and long patient queues are commonplace. Research can help shed light on these parts by highlighting aspects influencing communication.

## Background

The Swedish psychiatric care system is facing challenges such as increased waiting times for consultations and difficulty in recruiting and retaining staff [1]. Clinicians in all categories, including specialist physicians, nurses, and psychologists, are particularly affected. The competence level has declined due to retirements and failure to recruit new clinicians [1]. Turnover among new employees is high, resulting in a shortage of licensed clinicians, including specialist nurses [2]. This has led to a decline in nursing specialization in psychiatric care [3].

The care sector, in general, has developed into a complex and demanding work environment where financial goals often come before the medical values ​​of care, contributing to a cycle of stress and reduced quality of care.

Several studies have identified specific stress factors within the psychiatric profession, including heavier workloads, increasing administrative burdens and lack of staff and resources, violent patients, interpersonal conflicts, and ambiguities [4–6]. Recent research [7] on occupational therapists´ self-perceived organizational and social work environment in different work sectors showed that those working in psychiatric care in Sweden experienced the largest proportion of unfavourable working conditions due to high workload and increased stress, which can lead to risk factors for psychological problems and turnover intentions.

Communication in healthcare has been studied extensively, but recent studies have focused on miscommunication and the importance of communication for patient safety [8–10]. Effective communication between clinicians can be challenging due to interprofessional differences in education, language, roles, and power struggles [11, 12].

Communication is one of the psychiatric care’s core competencies [13]. Communication is the act of exchanging information between individuals through various methods such as language, images, gestures, and symbols. This process can be complex due to obstacles like non-verbal cues that can either strengthen or weaken the message [14]. Conversely, interaction refers to communication or reaction between two or more people or things, as the Cambridge Dictionary defines it. It involves actions that affect others and are reciprocated with a response, which can occur through gestures and language [15].

SBAR is a communication tool that provides short, organised, and predictable information for healthcare professionals, introduced in 2002 in Colorado, USA, to improve patient safety [16]. Interprofessional communication is important for safe patient care, as it allows different clinicians to share information and coordinate care effectively. It is recognized as a core competency for effective collaboration [9, 17, 18]. Interprofessional communication as a concept was further developed by Velásquez et al. [10], who described it as “the ability to communicate responsibly with patients and their families, health and other professionals, and the community”.

Previous studies on interprofessional communication have mostly been restricted to the relationship between physicians and nurses [12, 19–22], indicating that further research involving other clinicians within healthcare is needed. The present study aims to investigate the importance of interprofessional communication in an occupational group of different healthcare professionals (clinicians).

One fundamental factor for improving interprofessional communication is establishing a mutually positive and respectful relationship between colleagues, where an understanding of the role and competence of other professional groups is valued [9, 23]. Various clinicians approach clinical problems based on their own specific knowledge and ethical frameworks. Studies have shown that factors such as role appreciation can influence whether and how communication takes place. Effective communication between different clinicians in care may be challenging owing to interprofessional differences in training, language and roles [9, 12, 17].

Collaborative clinical relationships are considered beneficial for interprofessional communication. Gleeson et al. (2022) showed that collaborating with colleagues from different professions is important for interprofessional communication. In contrast, the study also showed that organizational factors, such as hierarchical work environments, constituted obstacles to interprofessional communication. Stressful work environments and excessive workloads can lead to miscommunication and unfriendly behaviour towards colleagues [9].

To achieve successful interprofessional teamwork, interprofessional communication is required [18]. Interprofessional teams work together with a shared identity to solve problems and provide services independently and in an integrated manner [24]. Team meetings are considered essential to interprofessional practice. Much of the work of interprofessional practice occurs outside formal information exchange processes, such as when professionals “take a moment” to give or receive information as they pass in the corridor—the quality of communication and how information is exchanged can contribute to creating tensions that affect interpersonal relationships. Communication is more likely to occur if the parties see a need to give or receive information from each other [17].

One significant part of an individual´s working life is the work environment. Interpersonal interactions affect nurses´ working life, and poor communication between clinicians may harm the organizational and social work environment [25–27].

The Swedish Work Environment Authority has regulations (AFS, 2015:4) that aim to promote health and prevent illness in the workplace. These regulations cover the organisational work environment, like management, communication, participation, and task allocation, as well as the social work environment, like collaboration and support from colleagues and managers [28].

In previous research, hierarchies, conflicts, and power struggles between nurses and physicians, in general, have been pointed out showing that communication between them can be seen as a work performance obstacle [12, 21, 22, 29]. At the same time, clinicians value each other’s perspectives in maintaining a healthy and good organizational and social work environment [9, 23]. Research has also shown that support from colleagues and supervisors can lead to a good organizational culture and improve communication between clinicians [30–32].

Because previous research has mainly focused on relationships between physicians and nurses in non-psychiatric environments [12], there is a gap in our theoretical knowledge concerning how interprofessional communication affects psychiatric clinicians´ organizational and social work environment. Previous research has predominantly been conducted in emergency, medical and surgical settings and shown that harmful and disrespectful relationships between physicians and nurses negatively affect the work environment [19, 20, 33].

The present study’s authors believe that more research on interprofessional communication between other clinicians is needed and that interprofessional communication studies should be explored from a broader perspective.

The present study aims to explore and describe interprofessional communication in a psychiatric outpatient unit. Furthermore, communication reflecting the organizational and social work environment can enable the development of hidden structures for interprofessional communication, which in turn can contribute to the strategic development of a positive organizational and social work environment and inclusive working life for psychiatric clinicians.

Through the ethnographic method, it is possible to understand the relationship between different clinicians and their interactions in practice. Ethnography distinguishes itself.

from other methodology sets after collecting empirical data in natural environments, i.e. those who do not get through interviews. Still, observations and interviews can result in discoveries that would not otherwise have been obvious, leading to deeper outcomes [34]. One potential drawback is that the researcher may become accustomed to their surroundings and lose objectivity from prolonged fieldwork, making reflexivity an important consideration.

## Aim

The present study aimed to explore and describe interprofessional communication in a psychiatric outpatient unit.

## Methods

### Design

The present study used a focused ethnographic approach inspired by Higginbottom [29] and Knoblauch [30] consisting of observations, informal conversations and formal semi-structured interviews. Focused ethnography involves, among other things, short-term visits but also intensive and extensive data. This approach makes it possible to understand the relationship between different clinicians and their interactions in practice. Through a subject-oriented focus on actions and interactions–, in the present study, interprofessional communication in the outpatient unit–, insights are gained into what the research intends to explore [35]. Fieldwork in the study was based on the first author’s (RN, specialised nurse in psychiatric care and PhD student) experience and was thus a subjective activity.

### Setting and participants

In Sweden, psychiatry became a medical specialty in the late 1850s [36]. Primary care handles mild to moderate mental illness, providing pharmacological treatment and psychosocial support. Severe cases are referred to specialist psychiatric care, which includes inpatient and outpatient services [37]. Outpatient care occurs at specialist clinics, hospitals, and municipal interventions like mental health centers, daycare centers, sheltered workshops, and clubhouses for individuals with mental disorders [38].

The outpatient unit provides mental health assessment, medical treatment, therapies, and support contact along with other treatments. Different clinicians, including psychiatrists, nurses (specialized and non-specialized), assistant nurses, psychologists, counsellors, physiotherapists, and occupational therapists, collaborate around the patient [37].

The study was conducted at a medium-sized psychiatric outpatient unit in central Sweden with a catchment area of approx. 1800 patients. Clinicians from various professions, including psychiatrists, nurses, both specialized in psychiatric nursing care and not, treatment assistants, psychologists, and more, were included in the study. Given the purpose of the study, no personal data was collected, but the clinicians mentioned in conversations and presentations that they all had previous experience in psychiatric care. The workplace unit in this study was predominantly staffed by women. For more information on the unit, see Table 1.

**Table 1:** Description of the essential features of the unit

**Place –** the physical place or places.	• The unit is L-shaped, and the staff have work rooms along two corridors. Everyone has their own office, and the manager’s office is at the far end of the shorter corridors. • The dining room is in the middle of the long corridor opposite the medicine room. • • Conference room B, found in the shorter corridor opposite the psychiatrists’ room, is the room used for most conferences; contains an oval table that fits about 10–12.
**Actor –** the people involved	• At this unit, about 25 people are employed, including treatment assistants, nurses, psychologists, psychiatrists, unit manager, medicine secretaries, curators, patient flow coordinators, and peer support. • The unit only has permanent employees; there are no temporary workers or hourly employees and no relay/hired staff. • Most clinicians had worked at the unit for a longer period, even when the working environment was alarming. Few were hired right at the end of that period; for example, the unit manager was hired when the report on an alarming work environment had just finished. • The clinicians are divided into different teams; based on diagnosis, patient group, and treatment, e.g., DBT (dialectical behaviour therapy) • Psychologists are responsible for psychological investigations and treatments, but other clinicians may also begin a neuropsychiatric investigation. • The treatment assistants are trained assistant nurses; they see patients, make assessments of new patients based on suspicion of psychiatric disorders, and administer rating scales to estimate neuropsychiatric or various anxiety and depression conditions. They provide support calls, do drug and treatment follow-ups, and document all work. • The nurses are further trained in psychiatric nursing. Their duties include, just like treatment assistants’ duties, follow-up and talk therapy but also medicine sharing and TeleQ (i.e., receiving calls when patients call in, but also checking messages via 1177 - Vårdguiden, a service from Sweden’s regions.) • The psychiatrists specialize in psychiatry and are responsible for diagnostics and drug treatment. They have the ultimate medical responsibility.
**Activity –** a set of related acts people do	• Treatment and reassessment conferences allow clinicians to agree on patient matters. At least one person from each professional group is present. These conferences are not divided based on which team the clinicians belong to. The clinicians go if they have a matter that needs to be dealt with in these conferences. • Regardless of profession, all clinicians on the unit assess the patient’s condition. • Specific assessments, e.g., medical history, interviews, and assessment scales, can be made with the patient, but interviews with relatives are also conducted. The assessments are made based on a referral for a suspected diagnosis • Team conferences are based on patient cases in the specific team. Team members participate.
**Time –** the sequencing that takes place over time	• Reassessment- and treatment conference on Wednesdays 13–17. • Team conferences on Thursdays. • Psychologist consultation time Tuesdays before noon. • The patient is enrolled in psychiatric outpatient care as long as his/hers condition requires specialist psychiatric care, with the option that the patient chooses to end the care contact.
**Object** – the physical thing that is present	• All clinicians are dressed in private clothes; some are more dressed up than others. • One psychiatrist wears scrubs (theatre blues - UK), while the other wears private clothes. • The unit’s code of conduct is often discussed and written on a whiteboard in the conference room. • There are name tags and a vacant/busy sign outside every office. Some clinicians always have the vacant sign visible, and some have the sign saying something between vacant and busy. Usually, the doors are open even when they are working on administrative assignments. • The clinicians select paintings in the unit from a predetermined selection chosen by a person in the region. The reception is bright and not clinically sterile. The coffee room has a large plant, sofa, armchairs and usually a fruit basket on the table.
**Goal –** the things people are trying to accomplish	• The overall goal is to put the patient’s needs first, provide the right help, and do this by working efficiently. • The code of conduct says that they should respect each other and each other’s opinions, listen to each other, cooperate and not be judgmental.

Selection of the psychiatric outpatient unit was based on expediency and geography, and none of the authors had any previous relationship with the setting or the clinicians. The inclusion criterion was that the outpatient unit must employ different clinicians. The exclusion criterion was that the unit must not be considered too small based on the number of employees (< 20 employees). Moreover, - staff who were not part of the care work (e.g., janitors) were excluded, as they were not involved in the regular daily communication in the psychiatric outpatient unit.

Initial contact with unit managers at the intended psychiatric outpatient unit was made via email, requesting the opportunity to come to the unit to inform them about the study. Once approval was obtained, the purpose of the study was presented in one digital meeting with the clinicians. Participant information was sent to their working email address before the follow-up meeting, in which all clinicians decided to participate. The first author presented the planned schedule for observations and gathered the signed consent forms in person. All individuals from the initial meeting attended the follow-up meeting, along with those who had not previously participated; these newcomers received pertinent information at the follow-up. Every clinician in the unit participated in the study, with no instances of declined participation. When new medical interns and residents joined during the observation period, they were briefed on the study and asked for consent before the observation session. Notably, there were no refusals to participate in any observation sessions.

### Data collection

Data collection was performed between May and September 2022. Data consisted of observations that included 100 h of fieldwork. Each observation session lasted approximately 4–5 h twice a week when various conferences were scheduled, and a multi-professional team participated. Field notes comprised about 46 pages, and reflection notes consisted of 14 pages. In addition to observations, six semi-structured interviews were conducted. Those who were available were chosen for interviews; based on that, they had different professions to some extent. For example, they were asked for an interview by the first author knocking on their office or asking them at coffee/lunch occasions. Of the respondents, all agreed and based on the clinicians´ work schedules, they decided when the interview would occur. Interviews were held in the interviewees’ office, lasting 30 to 80 min. Topics discussed included collaboration, workload, and responsibilities.

The interviews were audiotaped and transcribed verbatim to produce a readable document. All data were saved as de-identified audio files and de-identified transcribed text and stored so that unauthorised persons could not access them. Observation as a method helped in understanding the group’s cultural meanings and social structures and how these were linked to their organizational and social work environment. During the participant observations, interactions and communication were studied using an observation guide specially developed for the study. The first author participated as an overt participant observer, which meant ensuring close proximity to the clinicians in order to make them feel comfortable, thus counteracting the observer effect that might occur. The observations were mostly based on conferences where the first author was passive in the background and did not participate in the work/discussions, patient care, or nursing context. Observation notes were taken continuously, providing descriptions of the surroundings, actors, time, current events, and the observer’s reflections. Documentation was done through observation notes, reflection notes, audio files and with informal conversation, in other words, ordinary conversations and small talk, took place when the first author had observed something she wanted to have clarified, usually in direct connection when the opportunity to ask questions was given. These conversations were written down with supporting words and recorded when the opportunity arose.

### Data analysis

In focused ethnography, researchers iteratively challenged interpretations and generated insights through continuous data collection [39]. Data was collected and analysed non-chronologically, with the first author processing it based on daily reflections after each observation session. Audio files were transcribed and transcriptions were printed out. Following a few weeks’ break, where the first author mentally and physically separated from reading or writing about the material, all printed material was dealt with again. Highlights and notes were made based on the aim of the research. The analysis was a back-and-forth process between each step, e.g., between initial codes and emerging themes, but also cyclical as the data analysis process was ongoing and repeated [39, 40]. The first author initiated the analysis, which was continuously validated by all authors through discussion and reflection. The first author open-coded the data, basing the codes on the research aim to explore and describe interprofessional communication in a psychiatric outpatient unit—open coding involved directly and verbatim marking codes on transcripts. An example from the analysis can be seen in Table 2.

**Table 2:** Examples from the data analysis

Semantic units	Codes	Categories	Teams	
The nurse believes that there are classic symptoms but does not want to make a diagnosis herself.	Responsibility	Streamlining workflow	Workload Alone in responsibility	
“You get to make rating scales with the patient but can’t interpret them.“	New work assignments			
The psychiatrist raises his hand to speak.	Code of conduct	Structure and order	Leadership Hierarchy	
The nurse constantly turns to the psychologist and asks for his opinion.	Gives freedom but maintains order- Power			It affects interprofessional communication.
Now you can lift what is difficult and get heard and trusted.	“An open-minded atmosphere – climate”	Behaviours	A constant reminder lies over them like a wet blanket	
When her case is done, the nurse scrolls on her phone during the conference.	Laissez-faire			
We are a good unit and team; we take care of ourselves.	Acceptance of the situation and each other			

In the next step, to achieve validity, the first round of coding was presented to and discussed with the co-authors, all of whom are experienced qualitative researchers, to refine and shape the initial codes. After the code discussion, the first author proceeds with the analysis, iteratively processing the codes several times to identify key themes in the data [40]. Themes differed from codes in that themes were generally broader, and multiple codes could be used in a single theme. A composite text using Spradley’s grand-tour method [41] was written based on the data. New concepts and themes emerged through discussion among the authors at the same time as in-depth observations and interviews were conducted in the field. Themes were formed by re-reading the data and identifying similarities. Interviews validated observations and guided further data collection. The analysis was a back-and-forth process, and we sometimes had to backtrack based on new data. Data were collected by the first author, with subsequent discussions and analyses involving all authors for robust findings. Preliminary results were iteratively developed by all authors and refined by the first author to produce the final study results. Focused ethnography provided a comprehensive understanding of the unit’s culture, with observations reinforced through follow-up interviews and new perspectives leading to further observations, analyses, and results.

### Rigour

To ensure accurate ethnographic research, von Koskull [42] suggests fieldwork, spending prolonged time in the field, and sensitivity to language and cultural codes, and ensuring trustworthiness through credibility, transferability, and confirmation [43]. Trustworthiness in the present study, was achieved through triangulation of data, regular meetings among authors to analyse data, and collecting data over time until saturation was achieved. Another way to achieve trustworthiness was through member checking, where data, interpretations, and conclusions were tested with the participants from whom the data were initially obtained. Member checking was conducted with participants during ongoing analysis in October 2022.

To ensure the present study´s rigour, reflexivity was practiced. Reflexivity means critically thinking about interpretations and communication while staying open and reflective during research [42]. The first author has a background as a psychiatric nurse, and coming from the same profession can be a subtle factor if the researcher thinks she already understands everything. Through written and oral introspection, it was essential to make the first author aware of her social position, how the participants may perceive her, and how this may affect her ability to enter the field. As a field worker, she had to interact with what was being studied but also “realize” what was being learned and then pass that knowledge on in a suitable manner. Her presence could influence how and what the participants said and did. Therefore, reflexivity was an ongoing process throughout the study. Researchers not involved in the research process evaluated the study process and outcome, at the Academy´s research seminar, similar to what Lincoln and Guba [43]call an external audit.

### Ethical considerations

All participants were provided with written and verbal information, ensuring they understood the study goals and role of the researcher [44]. Consent was obtained from the operations manager and unit manager at the selected psychiatric outpatient unit. Participants were informed verbally and in writing that their participation was voluntary; they were free to withdraw from the study without consequences. The study received ethical approval from the Swedish Ethical Review Authority, which also assessed compliance with GDPR and that all methods were performed in accordance with the relevant guidelines and regulations. Because the unit is relatively small regarding the number of employees and because each profession group consists of one to a few individuals, citations in the results section will not describe which profession was cited; this was done to ensure the participants´ confidentiality.

## Results

The following results have emerged from observing the clinicians’ interaction and interprofessional communication and through analyses and supplementary interviews. Our results show how interprofessional communication within psychiatric outpatient units occurs daily.

**Fig. 1:**
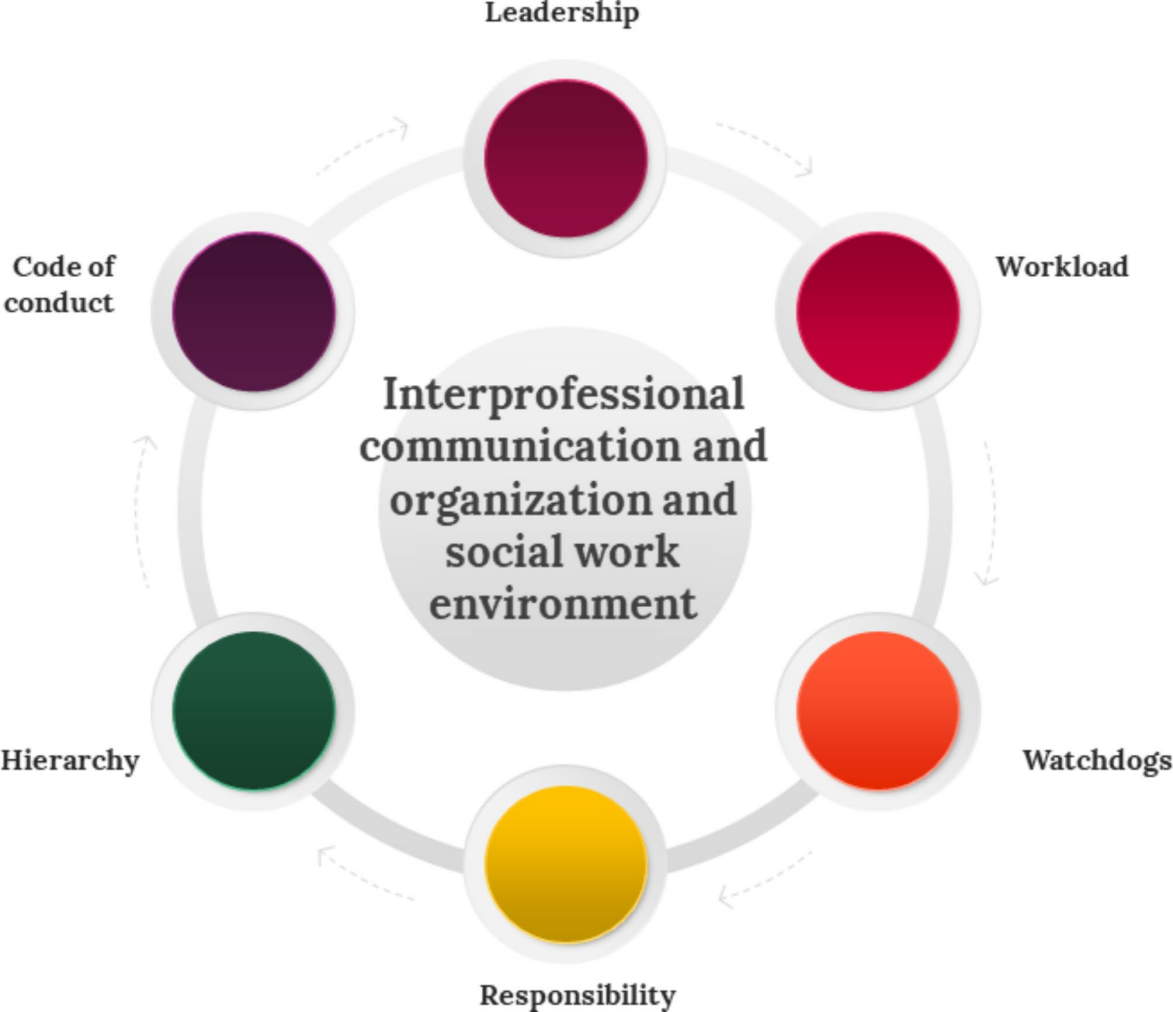
Influence of leadership, workload and responsibility, on the social and organizational work environment

Figure 1 highlights the impact of leadership, workload, responsibility, watchdogs, hierarchy and code of conduct on interprofessional communication and the social and organizational work environment. These elements have together significantly shaped the results.

To create a pre-understanding of the unit’s structure and function, Table 1 includes a description of the essential features, actors, activities, objects, time and goals of the place. See Table 1.

### “Reiterating the dark saga – a previous era of chaos and anarchy.“

At the very outset of data collection for the present study, the clinicians were eager to tell the unit´s history. They said that the unit had worked a great deal with the work group and the workflow, as there previously had been many problems and that it was much better now. That set the tone for a recurring history that the authors think has coloured the present. In the first observed conference, the unit manager and a psychiatrist sat at the computer to document and lead the conference. In the corner of the whiteboard, it was written: *“at all our unit meetings, we consider our code of conduct.“* The conference went on for a few hours at a leisurely pace, where the unit manager directed the meeting and the psychiatrist and psychologists interjected with occasional questions— the conference´s silence and the nature of its past were recounted in conversations with clinicians about structure.

Quotes about the unit’s history of discord, domineering techniques, and informal leaders were mentioned in discussions and interviews on several occasions.

After a while in the field, an apparent authority at the psychiatric outpatient unit was noticed. According to the first author´s interpretation, the unit manager showed authority by controlling and dictating the work process, by saying what must be done and how things should be pushed forward to promote efficiency and economy. However, the unit manager ensured that the clinicians felt involved in the unit’s work and were not excluded or overridden before closing a case; the psychiatrist or the unit manager asked the other conference participants for their opinion or if they wanted to add anything.

The unit manager or the psychiatrist held the reins in the different conferences and decided on further investigation, diagnosis, or other measures. On the other hand, if there was no psychiatrist or unit manager at the conferences, the clinicians seemed lost, and the structure and order of the conference fell apart. Because the clinicians seemed used to having an authority who was in control, the first author saw a lack of clear leadership when the unit manager and the psychiatrist were not there. No one else automatically took a leading role, but primarily a psychologist tried to keep the meeting going.

During this day´s conference, the first author draws attention to the fact that there is neither a unit manager nor psychiatrist on site, and in the reflection notes from the day can be read, *" The psychologists sit at the computer for documentation. There is no schedule on the board. The conference is perceived as unstructured; they get stuck on matters for a long time, there is no clear leadership, and everything takes a long time. The meeting ends, and there will be no treatment conference as the psychiatrists are not there. Again, the psychiatrists’ mandate to make decisions and the other’s dependence on them is reflected. Today, no one was so independent and sure of their cause that a case could proceed.”* – Fieldnote from April 20, 2023.

The psychiatrist was the one who ultimately decided how to proceed with a patient’s case, and the clinicians seemed relieved and satisfied with that. It seemed that when no psychiatrist or unit manager participated, the psychologists were next in line in the hierarchy, with the right to make decisions about treatment that nurses or treatment assistants did not have. In this way, the psychologists had the decision-making mandate when neither the psychiatrists nor the unit manager were on site. When clinicians were asked about the quiet conferences, the response was often, *“you do not know where we are coming from, historically.“* Because the clinicians repeatedly told the unit’s story, the first author believed they still feared that things could go wrong. This could happen if clinicians were not kept under control and reminded of the unit´s code of conduct, which they followed, and the unit´s story was seen as a reason not to question rules and working methods.

At a conference, the unit´s “watchdog” was mentioned, which was a way to keep order. The designation “watchdog” came from previous environmental work. By introducing watchdogs, acceptable and unacceptable behaviour at the conferences could be clarified. The “watchdog” job at conferences was to ensure that the schedule was followed, that the discussions were adequate for SBAR, that the perspectives of different clinicians were considered and that the tone was pleasant. “Watchdog” could be described as a working way to keep the clinicians and the subject at the conferences in order. However, it was used sparingly and not at all conferences. At conferences, the “watchdog” only sometimes fulfilled its intended function. On several occasions, discussions dragged on, SBAR was not followed, and other conference clinicians talked about other things while a patient case was being discussed. On these occasions, the watchdog did not speak up, which the first author interpreted as indicating that the watchdog’s purpose was just an automatic part of some conferences. The clinicians did not have free rein to manage their team meetings without constant supervision, which provided security for them to fall back on should the need arise.

The issue of constant supervision was raised in an interview with one of the clinicians that said, “*Historically, it was not a good work environment when I started here and then it was about whoever said something would decide, and the rest of us should do it. There was no hierarchy of physicians; more personable, informal leaders who had taken on the role and used domineering techniques that intimidated the rest of us, so many were afraid to speak up for fear of being attacked; every conference was like a Nuremberg trial. If someone had a different opinion then and tried to present it, it was an easy personal attack.” –* Interview in April 2023.

By appointing watchdogs and constantly highlighting the unit´s foundational values ​​and code of conduct, the clinicians were fed “reminders” to behave, the risk being that the group and the unit would otherwise fail and return to the chaos they constantly reiterated. It had become an imprinted mantra that no one seemed to reflect on. The workgroup had gone from chaos to order but with a constantly looming reminder of what could happen if they did not behave.

### Conditional responsibility

The clinician´s responsibility is to be able to cope with their tasks, which have increased in number over time, and at the same time be able to work in a group to ensure the best care for the patient. Responsibility can also be a matter of speaking up when one experiences something wrong or difficult. If the responsibility is conditional, the individual can take responsibility for his/her work to a certain extent. It also means coordinating with various professionals and thus getting “confirmation” that the work has been done correctly—responsibility but with limited freedom.

In the present study, clinicians were encouraged to take on new tasks and believe in their work, thus making the workgroup more effective. The unit manager encouraged clinicians to take responsibility, express their own opinions and trust their own judgments. There was an unspoken implication that they had to work independently to streamline patient management, and it was common for them to be responsible for diagnostic investigation and follow-up. The clinicians were seen and heard expressing that they were alone in this responsibility. Despite new tasks, psychiatrists had the ultimate responsibility for the patient, resulting in other clinicians having “free rein” to admit patients, lay the foundation for diagnostic assessment, and be encouraged to trust themselves in diagnosis and treatment. In teamwork with other clinicians, the psychiatrist often makes the final decision about the patient in a new assessment or treatment conference if the work has been sufficient and correct. Thus, in the end, it was a matter of individual clinicians with a great deal of responsibility for their patient case, having their work confirmed or reinforced by those with the mandate to make decisions, i.e., the psychologists and mainly the psychiatrists.

At the unit, it seemed as though everyone needed to do their part for the good of the unit, and because “everyone had a lot”, there was no one to turn to. Clinicians were encouraged to make their assessments, which could help them develop at the same time as this was precarious if the demands were too high and the threshold for asking for help was increasing. Feelings of high workload, many patients depending on few clinicians were expressed and seen in connection with conferences. Many “small matters” - such as continued sick leave or prescription renewal, took time and had to be squeezed in somewhere, as there was no time for it at the conferences. Clinicians from various professional groups were performing tasks that their professional group did not initially do, for example, diagnostic investigations. Still, achieving a better workflow had become necessary, sometimes requiring clarification about their new tasks and responsibilities.

A sense of being alone in their responsibility appeared mainly in nurses and treatment assistants, who had to carry out many investigations, make diagnoses, convey diagnoses, and follow up on drug treatment. Although several clinicians had told the first author they could always ask for advice and help, - they simultaneously experienced guilt and a reluctance to “burden” someone else with their thoughts. Instead, they tried to do everything themselves. The unit manager and psychiatrists encouraged other clinicians in different conferences to make a diagnosis if they had a good basis for it and to make their own decisions and carry out investigations. Most often, the clinician who reported on a patient case was asked, *“What diagnosis do you make?“* or received a comment, *“Then you write that diagnosis on your last journal entry.“* In interviews and conversations with the clinicians, they felt a great responsibility.

A quote from an interview revealed clinicians´ thoughts about workflow and responsibilities; *“Sometimes I think I need more skills for this. It is possible to get help and support, but it is not automatic; I have to ask for it, which is difficult. Who do I burden with this when everyone here has the same burden to whom should I delegate? Sometimes it can wake me up at night thinking, have I done the right thing? People often say this is too big and they cannot do it themselves and have to get help, but you cannot hand it over to someone who then has more to do just to make it easier for you. “*– From an interview in April 2023.

During interviews and in conversations, the first author asked clinicians about being personally responsible for patient cases at the conference and assessing the diagnosis; they responded:

*“It is getting to be too much, and no one can lighten the load. It is a great team, and I go to work with an open mind, but in outpatient care, you are very alone with responsibilities; even though we can and do work together, I have a big responsibility.“* - From an interview in April 2023.

Another answer was: “*On an individual level, some cope with the assessment process better, and some need more guidance than others. It is very clear when a clinician cannot say what diagnosis they are leaning towards; that tells me that that clinician does not know what she is doing. The idea is that the new assessment conference provides guidance on which direction to go based on the perspectives of different professions. In the best of worlds, the competence would be so high that we would not have to sit in conferences indefinitely. Some new assessment processes are very long due to lack of knowledge. However, in treatment conferences, this is ensured because different professionals discuss things together before anything is decided. That is our solution to making it good enough.”* - From an interview in October 2023.

On the unit, the manager and clinicians trust each other in their ability to speak up and ask for help. There is a pronounced feeling of being alone with responsibilities and an increased workload. At the same time, a fear of being a burden to someone else has been noticed, which can negatively affect interprofessional communication. One thought from the first author emerged during the study: efficiency and economics ruled to the extent that clinicians buried their heads in the sand regarding their work environment and instead hoped that the unit´s work would continue to flow.

### A hidden hierarchy in a permissive climate

In the present study, interprofessional relations, conversation, socializing and respect among the clinicians contributed to a relaxed and collegial atmosphere. Clinicians expressed openness and community within the workgroup. In various conversations and interviews with a number of clinicians, several of them expressed that this was their best workplace. Loud discussions and laughter were often heard in the lunchroom; the topics of conversation could vary from serious patient matters to clinicians´ privacy. One attitude the unit was perceived to have was that there should be an open and easy-going climate in the unit. Clinicians should feel welcome and be able to laugh and dare to express their opinions. At first glance, people were happy together and seemed equal in this workplace.

Although the unit had an open climate where all could laugh and joke across professional boundaries, there was still a reluctance to dare to express or question another professional’s opinion. At one meeting, clinicians were discussing and wanted to emphasize that even though psychiatrists and others had agreed that the patient’s current diagnosis was incorrect, they were not prepared to change the diagnosis, but instead discussed how to defend not going against the psychiatrist. During an informal conversation with clinicians about roles and questioning others’ opinions, especially a psychiatrist’s opinion, they answered: *“yes, I could certainly do that, but what is the point of it?“* In contrast, another clinician reported feeling able to discuss and express different opinions. The first author experienced, as mentioned earlier, the unit´s code of conduct as a practised mantra, where everyone says how good it is to have an open and pleasant climate. At the same time, fear and uncertainty existed, and they did not want to question too much. One clinician expressed a slight laissez-faire feeling where she agreed with the psychiatrist´s decision despite disagreement, because the patient would probably come back and be referred to the clinicians in question anyway.

Another clinician reported trying to make joint decisions and look at the case from different perspectives. Nevertheless, the psychiatrist usually had the final word, and sometimes it felt as though the psychiatrist neglected other clinicians´ perspectives. Even if the collaboration was perceived as good, there were times when clinicians disagreed and considered it was better to “go to bed” than to “argue” about it. However, if it was a clear case where other clinicians thought the psychiatrist´s perspective was “wrong”, they claimed they could speak up, but this was rarely observed during the present study.

Based on reflection notes from observation, it emerged that the opinions of different clinicians had different values. They reported believing that everyone was listened to, but changed their mind when the first author shared observations of this. They then agreed with the statement, but could not say why it had happened and that it has been like this at times before, but that it is not something they have noticed and discussed in the working group. At the workplace under study, clinicians dressed in personal clothes, except for a psychiatrist who dressed in theatre blues. The other clinicians’ answer to this was practicality, but the psychiatrist wearing theatre blues might also stand for something else. Taking a stand, hierarchy? At that claim, the clinicians only smiled and replied, *“who knows.“*

The clinicians in this psychiatric outpatient unit believed that hierarchy is usually more pronounced in inpatient care than in outpatient care. Although most clinicians did not experience a strong or negative hierarchy, opinions about hierarchy emerged in conversation with the first author: “*There is a clear hierarchy around who decides, and everyone having opinions is not always effective.“* – From an informal conversation in May 2023.

In summary, the working group could cooperate reasonably, have respect for each other´s professions and even have elaborate working methods for information exchange. However, teamwork and communication could be prevented by workload, insufficient resources and an underlying hidden hierarchy.

## Discussion

The present results reveal how a workplace´s history can impact interprofessional communication and the work environment, even long after changes have been made to improve it. Furthermore, the results show that clinicians´ professions, workload and responsibilities can affect interprofessional communication.

### Thriving to see better days

Psychiatric hospitals and cultures have been associated with, and criticized for, their hierarchy and obedience, power and socialization [45], features that other medical specialities have not been as prone to. Psychiatry has often been portrayed as a source of extreme cases of control and discipline, where clinicians exert control to maintain order in the psychiatric unit [46]. Based on the history of psychiatric care and its ruling hierarchies, it was of considerable importance to see that the psychiatric outpatient unit under study did not fit completely into that picture. Nevertheless, there was an underlying hierarchy—which is difficult to avoid given the overall organizational structure of healthcare—in which physicians held the highest position. Based on the roles of different professions, we observed thriving for equality, where everyone’s opinions were important and interprofessional communication was a cornerstone of work at the unit. In the absence of respect for the equal value of all clinicians, the hierarchy could create interprofessional communication barriers, which were occasionally observed in the form of “wait-and-see behaviour”. Similar behaviour has been previously described to have devastating outcomes. For example, in the case of Elaine Bromiley, a healthy woman who died because two anaesthetists failed to intubate during routine surgery, two of the nurses involved knew what to do but did not assert themselves because of the hierarchy. They instead used passive and indirect statements that were ineffective during the crisis [47]. In our results, this could be seen in the fact that some of the clinicians did not challenge the hierarchical structures and questioned the psychiatrist´s diagnosis but waited with a conviction that the patient would still come to them at a later stage.

Our results show a high workload caused by new responsibilities and nurse shortages. Previous studies [4, 9]have shown that these factors are associated with the risk of poor teamwork and interprofessional communication as well as with identified stress factors, especially in psychiatry, such as increasing administrative burdens and lack of resources [4–6], as seen in our results. To deal with the workload at the unit, the clinicians have tried delegating work tasks. Interprofessional communication involves responsibility and trust in clinicians’ ability to perform new tasks. Trust in an individual´s ability to perform tasks is also rooted in role understanding and respect for each other´s professional roles, as shown in prior research [9, 25], which are key to promoting good interprofessional communication and collaboration. Moloney et al. [48] described how friendly and professional interactions foster a climate of trust and respect, resulting in a successful and clear organizational vision that leads to a positive work life.

Interprofessional communication supports responsibility development and coping with work tasks, as seen in the Swedish Work Environment Authority’s regulations [28]Streamlining work tasks, like delegating diagnostic examinations, can empower clinicians, but it may prioritize efficiency over workgroup relationships, increasing workload. Clinicians may find it difficult to ask for help in such situations.

Much of healthcare involves the efficient use of resources [49]. In healthcare, efficacy means potential treatment effectiveness under ideal conditions, while efficiency is about economical approaches [49]. Our results show that clinicians handle new tasks efficiently, but they also face high workloads and uncertainty.

Today, the unit is trying to achieve a work environment in which the workflow during conferences is more efficient. Through the unit´s code of conduct and watchdogs, — prompted by the unit´s past—supportive and clear leadership guide the unit´s work forward. Efficiency reigns, but how different clinicians´ experiences and having a respectful tone between clinicians promote continued thriving and collaboration in the work environment is highlighted. Interprofessional communication is an important part of psychiatric outpatient work, where efficiency, insufficient staffing and long queues to patient care are common. Efficiency may lead to neglect of nursing itself. Psychiatric nursing work has been considered invisible and lacks role clarity [50], which is also evident in our study. The priority was diagnosis and treatment and streamlining the workflow to shorten the queues based primarily on the medical perspective, as the psychiatrist had the final say.

Hierarchy always presupposes a ranking of values and unequal social power or status [51]. In healthcare systems, physicians are given the control position and play a leading or decision-making role [52]. The term hierarchy often has a negative connotation and can entail placing those with less power in a subordinate and dependent position [53]. Nevertheless, hierarchy creates a kind of recognition structure for the healthcare profession because clinicians are trained in different tasks depending on their respective professions [17].

Our findings question contemporary psychiatric hospital cultures regarding obedience, control, and power positions (cf. 37,38). We argue that our results give support for destigmatization processes promoting a change in attitudes and behavioural intentions that promotes thriving. However, the change was not a spontaneous automatic process. Rather, drawing on complexity theories, changes in open systems, i.e. human cultures, presuppose an input [54]. Thus, the input was staff exertion, in which the code of conduct and leadership qualities played a significant role. Manojlovich et al. [55] stated that establishing a code of conduct improves interprofessional communication. Interpretations based on the collected data show that the code of conduct perpetuated ideas about clinicians’ behaviour, leading to a thriving work environment manifested in conferences transitioning from “Nuremberg trials” to respectful discussions.

Improving interprofessional communication is more than “appreciating someone´s opinion.“ When diagnoses and treatment options are discussed at conferences from a medically-centred dominance without the input of nurses, we interpret that their professionalism is not fully recognized. Nurses can assertively express their evaluations and recommendations using standardized nursing terminologies, contributing to a more equal and collaborative environment. Using these terms helps nurses advocate for their patients and improve the quality of care, strengthening the nursing profession [56, 57].

The conferences at the unit contribute to cross-border work between the different clinicians. They allow collaboration and getting help from others when clinicians sometimes feel unsure about, e.g., the interpretation of rating scales. Manojlovich et al. [55] showed that physicians and nurses have different perspectives on the same clinical situation, which affects the perception of what is important or urgent. This could be identified as aspects of responsibility and workload, as shown in our results, where ‘small cases’ were not dealt with, and cases brought up in conferences were sometimes sent back to a nurse to sort out. Juggling multiple tasks simultaneously and being the “spider in the web” takes time away from nursing work, especially when the nurse has to serve other clinicians in the team and put her patient and ongoing care tasks on hold [58].

### A quest for a work environment in balance

Today Swedish psychiatric care is struggling greatly to change the image of psychiatry. At the same time, it is an area with difficulties recruiting new staff. The number of individuals with mental illness is increasing in society [59] and the media’s depiction of psychiatry is largely negative [60].

Interprofessional communication strategies differ significantly between team members, professional categories and assignments [61]. Our study shows communication methods varied based on the context. The dining room had an open and relaxed climate, with no clear boundaries on what was said. In conferences, communication was more tentative, depending on the conference leader, case nature, and workload.

There are reasonable grounds for assuming that interprofessional communication is related to and influences collaboration, which some research supports [12, 62]. However, this remains speculation, as collaboration was not an area studied per se in this research. Further research is needed on how interprofessional communication and collaboration are related.

The present results show a unit that strives to improve interprofessional communication and safety work around patients through changed tasks to meet today´s care queues. Clinicians face organizational facts such as reorganizations and low staffing, while the number of patients needing specialized psychiatric care is increasing. Research can help shed light on this by highlighting factors that influence interprofessional communication.

### Methodological considerations

Inspired by focused ethnography, the method was data intensive and characterized by selected, specified, focused aspects of a field [35, 39]. The method provided an opportunity to learn more about culture, group behaviours and/or group interactions and increased understanding of human behaviour, thus strengthening the study. When certain aspects of a culture or environment become routine, most participants are no longer aware of their behaviours or actions. In qualitative research, data collection tends to include “triangulation”, which refers to the use of multiple data collection methods. The present study used, e.g., semi-structured interviews with observation to compare data and confirm findings [39]. One methodological challenge was maintaining a neutral frame of mind and avoiding bringing personal bias, based on one´s own lived experience, into the equation, —which could affect reliability. However, to take in and gain access to the personal experiences of a culture in action, a certain commitment on a personal level must exist. The methodological advantages are the participants´ openness and honesty; a possible drawback is their awareness of being observed, which may lead them to present themselves in a way that would not otherwise come naturally to them [34]. Given the purpose of the study, no personal data was collected, which can be seen as a limitation as no additional information about the number of years of work in psychiatry or further education was disclosed. Another limitation of this study is that it was performed at only one outpatient unit, which makes it more difficult to see the results as general for psychiatric outpatient care.

## Conclusions

Our results indicate that leadership, an inclusive working style, and an open environment— where speaking up is encouraged and valued— can be beneficial in fostering interprofessional communication and collaboration. Clinicians having respect for each other’s professional roles is key to achieving this.

An appetizing avenue for future research lies in exploring the role of socio-spatial places within psychiatric outpatient units. These places include physical spaces and social interactions that shape patient, clinician, and staff relationships. Interdisciplinary conferences have been identified as a crucial mediating tool that grants power to both the subjects and clinicians, positioning the latter as agents of power. Despite the challenging workload, maintaining interdisciplinary communication is a priority, and introspective contemplation is being used to facilitate communication. Researchers could gain valuable insights into enhancing patient-centred care and achieving positive outcomes by delving into the dynamics of socio-spatial places in psychiatric outpatient units. *“Not only do people make spaces, but spaces may be used to make people " (Halford & Leonard, 2003:202).*

## Data Availability

The dataset generated and analysed during the current study are not publicly available due to participants’ confidentiality and the researchers have no ethical permission to share them. The corresponding author can be contacted if someone wants to request the data from the study.
